# Probiotic Consortium Confers Synergistic Anti-Inflammatory Effects in Inflammatory Disorders

**DOI:** 10.3390/nu16060790

**Published:** 2024-03-11

**Authors:** Changhon Lee, Seung Won Kim, Ravi Verma, Jaegyun Noh, John Chulhoon Park, Sunhee Park, Haena Lee, Hye Eun Park, Chan Johng Kim, Seohyun Byun, Haeun Ko, Seungyeon Choi, Inhae Kim, Soomin Jeon, Junglyoul Lee, Sin-Hyeog Im

**Affiliations:** 1 Department of Life Sciences, POSTECH Biotech Center, Pohang University of Science and Technology (POSTECH), Pohang 37673, Republic of Korea; changhon_lee@hms.harvard.edu (C.L.); kso251@postech.ac.kr (S.W.K.); jgnoh96@postech.ac.kr (J.N.); johnchpark@postech.ac.kr (J.C.P.); hnlee16@postech.ac.kr (H.L.); parkhyeeun@postech.ac.kr (H.E.P.); chanjkim@uw.edu (C.J.K.); shbyun77@postech.ac.kr (S.B.); haeunko@postech.ac.kr (H.K.); 2 ImmmunoBiome Inc., 77 Cheongam-ro, Nam-gu, Pohang 37673, Republic of Korea; raviverma@jnu.ac.in (R.V.); psh@kist.re.kr (S.P.); seungyeon1320@gmail.com (S.C.); ihkim@immunobiome.co.kr (I.K.); 3 hy Co., Ltd., 22 Giheungdanji-ro 24 beon-gil, Giheung-gu, Yongin 17086, Republic of Korea; 10003273@hy.co.kr; 4 Institute for Convergence Research and Education, Yonsei University, Seoul 03722, Republic of Korea

**Keywords:** probiotic consortium, microbiota, atopic dermatitis, regulatory T cell, inflammatory colitis

## Abstract

The composition and diversity of gut microbiota significantly influence the immune system and are linked to various diseases, including inflammatory and allergy disorders. While considerable research has focused on exploring single bacterial species or consortia, the optimal strategies for microbiota-based therapeutics remain underexplored. Specifically, the comparative effectiveness of bacterial consortia versus individual species warrants further investigation. In our study, we assessed the impact of the bacterial consortium MPRO, comprising *Lactiplantibacillus plantarum* HY7712, *Bifidobacterium animalis ssp. lactis* HY8002, and *Lacticaseibacillus casei* HY2782, in comparison to its individual components. The administration of MPRO demonstrated enhanced therapeutic efficacy in experimental models of atopic dermatitis and inflammatory colitis when compared to single strains. MPRO exhibited the ability to dampen inflammatory responses and alter the gut microbial landscape significantly. Notably, MPRO administration led to an increase in intestinal CD103^+^CD11b^+^ dendritic cells, promoting the induction of regulatory T cells and the robust suppression of inflammation in experimental disease settings. Our findings advocate the preference for bacterial consortia over single strains in the treatment of inflammatory disorders, carrying potential clinical relevance.

## 1. Introduction

Commensal microbes ubiquitously inhabit the human body, profoundly influencing various physiological functions such as immune modulation, digestion, and behavioral patterns [1,2]. Particularly in the gut, imbalances in the intestinal microbiota, known as dysbiosis, are closely linked to the etiology and progression of diseases, including inflammatory autoimmune and allergic disorders [3,4]. Consequently, targeting the gut microbiota has emerged as a promising therapeutic avenue for immune-mediated disorders.

Over the decades, numerous studies have highlighted the potent role of specific bacterial strains in modulating host immunity and mitigating excessive inflammatory responses [5,6]. For example, *Bifidobacterium bifidum* has been shown to induce the differentiation of regulatory T (Treg) cells and enhance their suppressive capabilities within the intestinal tract [7]. Moreover, bacterial consortia, characterized by the combination of two or more bacterial strains, have exhibited significant immunomodulatory potential, ameliorating various experimental models of inflammatory, autoimmune, and allergic disorders [5,8,9,10]. For instance, IRT5, a blend of five probiotic strains, has demonstrated immunosuppressive properties in conditions such as atopic dermatitis, rheumatoid arthritis, myasthenia gravis, and multiple sclerosis [11,12]. However, comprehensive comparative studies evaluating the suppressive capabilities of individual bacterial strains versus consortia are limited, offering restricted evidence on their efficacy and underlying mechanisms [13,14,15,16]. The optimal strategies for developing microbiota-based therapeutics, particularly the advantages of single strains versus bacterial consortia, remain a subject of ongoing debate.

Atopic dermatitis (AD) is a prevalent chronic inflammatory skin condition characterized by recurring episodes, with its incidence steadily rising in recent decades [17,18,19]. The multifaceted nature of AD involves genetic, environmental, immune dysfunction, and the dysbiosis of commensal bacteria [18]. AD patients display diverse symptoms such as dry skin, pruritus, localized eczema, and elevated immunoglobulin E levels [19]. Previous studies underscore the pivotal role of T helper (Th2) cells in AD pathogenesis, alongside contributions from other T helper cell subsets, including Th1 and Th17 cells [18,20]. Notably, individuals with AD exhibit gut microbiota dysbiosis, exacerbating the condition by promoting harmful inflammatory responses [21]. Interventions targeting the microbiota have garnered attention in both preclinical and clinical studies, yet effective microbiota-based therapeutics for AD remain elusive [22].

Inflammatory Bowel Disease (IBD), a chronic gastrointestinal inflammatory disorder, affects approximately 4.9 million individuals worldwide as of 2019, with its global prevalence continuously escalating [23,24]. Decades of research elucidate the complex interplay between genetic and environmental factors contributing to IBD pathogenesis [24,25]. The gut microbiota significantly influences IBD pathophysiology, prompting extensive studies aiming to develop efficacious therapeutics by targeting the gut microbiota [26]. Although numerous studies have identified diverse bacterial strains with immunomodulatory capabilities showing promise in preclinical studies with experimental IBD models, no microbiota-based therapeutics have gained approval from the U.S. Food and Drug Administration (FDA) to date. The lack of an adequate strategy for developing microbiota-based therapeutics, along with limited evidence on the comparative efficacy of single immunomodulatory bacterial strains versus bacterial consortia, contributes to this challenge.

This study comprehensively compares the beneficial effects of a bacterial consortium to its individual component strains, focusing on the MPRO consortium composed of *Lactiplantibacillus plantarum* HY7712, *Bifidobacterium animalis* ssp. *lactis* HY8002, and *Lacticaseibacillus casei* HY2782. Through systematic investigations in both healthy and diseased mouse models, we assessed the immune-modulating capacities of MPRO and its individual strains. While the individual strains exhibited relatively limited efficacy against AD, the MPRO consortium displayed significant potential in alleviating inflammatory symptoms in AD. This consortium regulated the immune system and rectified gut dysbiosis. Additionally, our findings confirmed the stronger efficacy of the bacterial consortium in alleviating experimental inflammatory bowel disease. These results strongly support the notion that utilizing bacterial consortia enhances synergistic immune-regulatory effects, both in steady states and disease-associated inflammatory conditions. This emphasizes the importance of employing bacterial consortia as the optimal approach to enhance outcomes in developing novel microbiota-based therapeutics for inflammatory disorders.

## 2. Materials and Methods

### 2.1. Mice

C57BL/6 (B6) and BALB/c mice were maintained under SPF conditions (The Jackson laboratory, Bar Harbor, ME, USA) in the animal facility of the POSTECH Biotech Center in accordance with institutional ethical guidelines. Congenic *CD45.1^+^* mice and *Foxp3-eGFP* mice were obtained from the Jackson Laboratory. *Rag1^−/−^* mice were obtained from Taconic. Gender- and age-matched mice between 5–8 weeks old were used. Germ-free C57BL/6 (GF B6) mice were kindly provided by Drs. Andrew Macpherson (Bern Univ., Switzerland) and David Artis (Univ. Pennsylvannia, USA) and maintained in sterile flexible film isolators (Class Biological Clean Ltd., Madison, WI, USA) in the animal facility of the POSTECH Biotech Center under institutional ethical guidelines. Gender- and age-matched mice between 6–8 weeks old were used. All mice were cared for under IACUC guidelines from the animal facility of the POSTECH Biotech Center. To determine the sample size for the animal experiments, we conducted pilot experiments and utilized the Sample Size Calculation resources provided by Taconic Biosciences “https://www.taconic.com/resources/animal-research-sample-size-calculation (accessed on 28 February 2024)”. Employing a power level of 80% and *p* = 0.01, we calculated the minimum number of mice required for each respective in vivo study. Additionally, to account for potential technical issues during disease induction and progression, we included 1 or 2 extra animals. We referenced our previous studies to determine the optimal dosage of probiotics administration in our in vivo efficacy studies.

### 2.2. Probiotic Strains

*Lactiplantibacillus plantarum* HY7712, *Bifidobacterium animalis* ssp. *lactis* HY8002, *Lacticaseibacillus casei* HY2782, and the bacterial consortium MPRO used in this study were provided by hy Co., Ltd. (Yongin, Republic of Korea). The bacterial consortium MPRO consists of HY7712, HY8002, and HY2782. To assess the effects of individual bacterial strains or MPRO, mice were orally given either single bacterial strains or MPRO daily, at a concentration of 5 × 10^8^ CFUs per 200 μL. The dose of probiotics for in vivo studies was determined on the basis of data from the previous studies [11,12].

### 2.3. Colonization of Bacteria in Germ Free Mice

GF mice were mono-colonized with single strains of bacteria or MPRO in separate isolators by administering bacterial concentrations of 1 × 10^9^ CFUs per 200 μL. Initial colonization checks were performed two weeks after administration by collecting fecal pellets from each isolator. Bacterial genomic DNAs were isolated from fecal pellets or luminal contents using the NucleoSpin DNA Stool (Macherey-Nagel, Düren, Germany) and confirmed by 16S rRNA PCR analysis. The following primer sets were used: total bacteria, EUB (forward) 5′-TCCTACGGGAGGCAGCAGT-3′ and (reverse) 5′-GGACTACCAGGGTATCTAATCCTGTT-3′ PCR analysis was carried out using a C1000 Touch Thermal Cycler (BioRad, Hercules, CA, USA).

### 2.4. Isolation of Lymphocytes and Flow Cytometry Analysis

Small intestines and colons were opened longitudinally and washed with PBS to remove mucus and feces. Intestines were cut into 1 cm pieces, and the portions were incubated and stirred with magnetic bars in PBS containing 10 mM EDTA, 20 mM HEPES, 1 mM sodium pyruvate, and 3% FBS for 20 min at 37 °C. The portions were washed with PBS and minced into smaller pieces and incubated in an RPMI 1640 media containing 3% FBS, 20 mM HEPES, 1 mM sodium pyruvate, 0.5 mg/mL of Collagenase D (Roche, Basel, Switzerland), and DNase I (Sigma, Mannheim, Germany) while stirring with magnetic bars for 45 min at 37 °C. The tissues were incubated for an additional 5 min in the presence of 10 mM EDTA. The soup was filtered into ice-cold PBS over 100 mm cell strainers. Cells were put over a Percoll^TM^ (GE Healthcare, Uppsala, Sweden) gradient (40% percoll on top, 75% percoll on the bottom) and spun at 2000 rpm for 20 min with no brake. The cells at the interface of the 40% and 75% layer were taken and washed twice with RPMI media supplemented with 10% FBS (Hyclone, Australia) and 1% Penicillin/Streptomycin and used for FACS staining.

Ears were cut into four pieces, and the portions were incubated and stirred with a magnetic bar in PBS containing 10 mM EDTA, 20 mM HEPES, 1 mM sodium pyruvate, and 3% FBS for 20 min at 37 °C. The portions were washed with PBS and minced into smaller pieces, and incubated in a RPMI 1640 media containing 3% FBS, 20 mM HEPES, 1 mM sodium pyruvate, 1 mg/mL of type V collagenase (Sigma Aldrich, St. Louis, MO, USA), and DNase I (Sigma, Mannheim, Germany) while stirring with magnetic bars for 45 min at 37 °C. The soups were filtered over a 100 mm cell strainer and used for FACS staining. Naïve CD4^+^ T cells (CD45.1^+^, CD4^+^, Foxp3^−^, and CD45RB^hi^) were isolated from spleens, pLNs, and mLNs using an FACS sorter (Astrios, Beckman Coulter, Brea, CA, USA).

### 2.5. Flow Cytometry Analysis

For live/dead staining, single-cell suspensions from mesenteric lymph nodes, small intestines, colons, and ears were stained with Fixable Viability Dye (Invitrogen, Carlsbad, CA, USA). For surface marker staining, cells were washed with PBS and stained with the following antibodies: CD45 (30-F11), CD45.1 (A20), TCRβ (H57-597), MHCII (M5/114.15.2), CD11b (M1/70), B220 (RA3-6B2), CD4 (GK1.5, RM4-5), CD8a (53-6.7), CD11c (N418), Ly6C (HK1.4), Ly6G (1A8), Siglec F (E50-2440), CD103 (2E7), CX3CR1 (SA011F11), CD317 (927), Nrp1 (3E12), CTLA-4 (UC10-4B9), and CD45RB (16A). For intracellular transcription factor staining, cells were fixed with eBioscience/Invitrogen Foxp3 Fix/Perm Buffer washed with eBioscience/Invitrogen Perm Buffer and stained with the following antibodies: Foxp3 (FJK-16s), T-bet (eBio4B10), GATA-3 (16E10A23), RORγt (B2D), and Helios (22F6). For intracellular cytokine staining, two methods were used. (1) Cells were restimulated by 100 ng/mL PMA (Calbiochem) and 500 ng/mL of ionomycin in the presence of Golgi-Plug (BD Biosciences, 555029, 0.5 μL/sample) for 5 h. (2) Cells were restimulated with Cell Stimulation Cocktail plus protein inhibitors (00-4975-03, eBioscience/Invitrogen, Carlsbad, CA, USA). After restimulation, cells were washed, and surface markers were stained. Cells were then fixed with eBioscience/Invitrogen Foxp3 Fix/Perm Buffer or eBioscience/Invitrogen Intracellular (IC) Fixation Buffer, washed with Perm Buffer and stained with the following antibodies: IFN-γ (XMG1.2), IL-10 (JES5-16E3), IL-13 (eBio13A), IL-17A (TC11-18H10.1), IL-17A (TC11-18H10.1), and IL-4 (11B11). A comprehensive list with detailed information on the source, clone, format, and catalog number of all the antibodies is provided in Supplementary Table S1. Cell acquisition was performed using an LSR Fortessa flow cytometer (BD Biosciences, Franklin Lakes, NJ, USA) or a CytoFLEX flow cytometer (Beckman Coulter, Brea, CA, USA) at the Microbiome Core Facility of POSTECH. Data were analyzed using FlowJo software (v10.9.0, Tree Star).

### 2.6. Induction of Experimental Atopic Dermatitis 

BALB/c were sensitized by painting each ear with 20 μL of 1.5% of 2,4-Dinitrochlorobenzene (DNCB) (TCI, C0162) dissolved in acetone/olive oil solution (acetone/olive oil = 1:3) at Day 0. After 7 days of sensitization, each ear was painted 2 times per week with DNCB or HDM extract repeatedly for 6 weeks. First, mice were challenged with 20 μL of 1% DNCB, dissolved in acetone/olive oil solution (acetone: olive oil = 1:3), in each ear. After 4 days of DNCB painting, 20 μL of 10 mg/mL HDM extract (Greer, Lenoir, NC, USA, XPB81D3A25) in 0.5% Tween-20/PBS solution was repainted. After 12 h of every challenge, ear thickness and clinical symptoms were monitored.

### 2.7. Histology 

Ear tissues were collected and fixed in 4% paraformaldehyde solution at 4 °C overnight. Tissues were embedded in paraffin blocks, sectioned at 3 μm thickness, deparaffinized, and dehydrated via sequential addition of xylene, 100% ethanol, and 95% ethanol. The sections were washed in distilled water and stained with Hematoxylin (HHS32, Sigma, St. Louis, MO, USA) and Eosin (HT110132, Sigma, St. Louis, MO, USA). Sections were imaged using a LEICA DFC420 C light microscope.

### 2.8. Induction of Experimental Colitis through Adoptive Transfer of CD4^+^ T Cells

FACS-sorted CD4^+^Foxp3^−^CD45RB^hi^ naïve T cells (1 × 10^6^) from congenic CD45.1^+^ Foxp3-eGFP mice were intravenously transferred to *Rag1* deficient mice. Recipients were orally administered with mock, HY7712, HY8002, HY2782, or MPRO every other day during the entire experimental period. Mice were sacrificed when their body weight decreased to 20% of their initial weight. Disease severity was analyzed by measuring colon length and cytokine production from donor CD4^+^ T cells.

### 2.9. Bacterial 16S rRNA Sequencing

A total of 30 mouse fecal samples for 6 groups (Mock, Vehicle, HY7712, HY8002, HY2782, and MPRO) before sacrificing were processed for DNA extraction using a DNeasy PowerSoil Kit (Qiagen, Hilden, Germany) following the manufacturer’s instructions. Samples were further processed for amplicon (V3–V4 region). Each sample was prepared according to the 16S Metagenomic Sequencing Library Preparation Part #15044223 Rev. B. Microbiome library was generated with the Herculase II Fusion DNA Polymerase Nextera XT Index V2 Kit, followed by metagenomic sequencing on an Illumina Miseq platform. The raw reads were processed using DADA2 to obtain the amplicon sequence variants (ASVs). Biological diversity analyses were performed using the physeq package of R software (v1.46.0). Unweighted UniFrac and weighted UniFrac were used to calculate beta diversity, and the calculated distance was expressed as PCoA. Pairwise permutational multivariate analysis of variance (PERMANOVA) was used to test the significance of differences in beta diversity in each pair of samples. After the PERMANOVA test, differences between groups were tested by FDR correction. The significance level Kruskal–Wallis analysis was performed to check whether the microbial ratio of each group was different, and the Scheffe method was performed as a post-analysis (significant level 0.05). The comparison of microbial ratios for each group was performed at the phylum and genus and was shown in the cumulative bar graph. The comparison of atopic-related strains was compared by indicating the proportion of each microorganism as a boxplot.

### 2.10. Statistical Analysis

Statistical analysis was performed with GraphPad Prism software (v10. 2.0, La Jolla, CA, USA). Differences between control and experimental groups were evaluated using two-tailed unpaired-Student’s *t*-test. Data are presented as mean ± SEM.

## 3. Results

### 3.1. MPRO Consortium Exhibits More Immunoregulatory Potential Compared to Individual Bacterial Species

**Figure 1:**
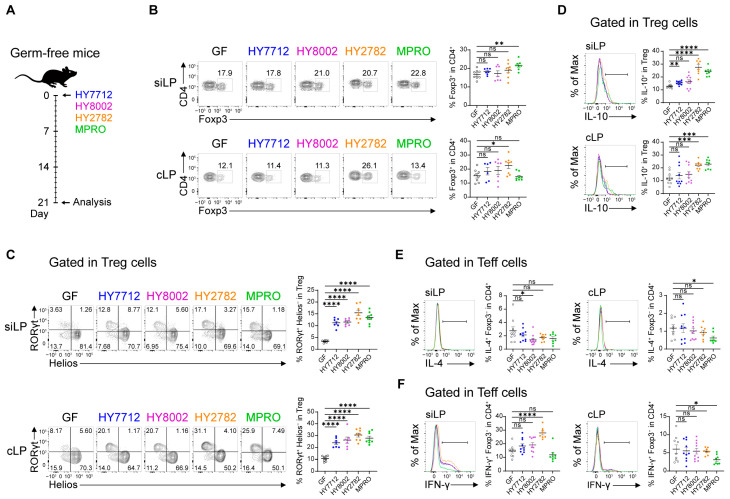
Bacterial consortium (MPRO) surpasses its individual components in immunoregulation. (**A**) Experimental scheme: GF C57BL/6 mice were colonized with HY7712 (blue), HY8002 (magenta), HY2782 (orange), or MPRO (green) for 3 weeks. GF mice were presented with black color open dots. (**B**) Flow cytometry plots and frequencies of Foxp3^+^ cells in CD4^+^ T cells in the small intestine (siLP) and colon (cLP) of indicated mice. Representative flow cytometry plots and frequencies of RORγt^+^Helios^−^ (**C**) or IL-10^+^ (**D**) cells within the Treg cell population. Representative flow cytometry histograms and frequencies of IL-4^+^ (**E**) or IFN-γ^+^ (**F**) cells within the Teff cell population (CD4^+^Foxp3^−^). Each dot represents an individual mouse. The graphs show the mean ± SEM. (**B**–**F**) * *p* < 0.05, ** *p* < 0.01, *** *p* < 0.001, **** *p* < 0.0001 (Two-tailed unpaired-Student’s *t*-test). ns: not significant. Data were pooled from two to three independent experiments.

We initially investigated whether the immunomodulatory potential of MPRO differed from that of its individual component strains by characterizing their immunophenotype. For this purpose, Germ-free (GF) mice were colonized individually with HY7712, HY8002, or HY2782 bacteria, as well as the MPRO consortium, all containing equal colony forming units (CFU) of the three bacteria (Figure 1A and Figure S1A). The examination of immune profiles in the small intestinal lamina propria (siLP) and the colonic lamina propria (cLP) revealed marginal alterations in the frequency of CD4^+^Foxp3^+^ regulatory T cells (Tregs) between GF mice and those treated with HY7712 and HY8002 (Figure 1B). HY2782-colonized mice exhibited a significant increase in the Treg frequency in the colon, but not in the small intestine (Figure 1B). Mice colonized with HY2782 showed a significant increase in the Treg frequency in the colon, but not in the small intestine (Figure 1B). Interestingly, MPRO treatment significantly elevated the Treg frequency in the siLP, but not the cLP (Figure 1B). While the frequencies of Tregs were altered only in HY2782 and MPRO colonized mice, bacteria-treated mice exhibited a substantial increase in the bacteria-induced RORγt^+^ Helios^−^ Treg cell compartment in both the small intestines and colons, regardless of the strain (Figure 1C). The functional analysis of Tregs revealed that HY7712 administration could moderately increase IL-10 expression in the siLP, while HY2782 and MPRO significantly upregulated it in both the siLP and cLP (Figure 1D). Additionally, MPRO colonization led to a significant reduction in inflammatory signatures such as IL-4 and IFN-γ in effector CD4 T cells (Teffs) from the cLP (Figure 1E,F). Although not statistically significant, IL-4 and IFN-γ expression levels in the Teffs also exhibited a trend toward a decrease in the siLP following MPRO administration (Figure 1E,F). Specifically, within the siLP, HY8002 was the sole strain significantly reducing IL-4, while HY2782 markedly increased IFN-γ among Teffs (Figure 1E,F). Within the mesenteric lymph nodes (mLN), the frequencies of Tregs were similar in all groups (Figure S1B). However, a closer analysis of RORγt and Helios expression revealed an increase in RORγt^+^ Helios^−^ Tregs in the mLN across all bacteria-treated groups (Figure S1C). These findings collectively suggest that although individual bacterial species may possess some immunomodulatory potential, a consortium of all these bacteria exerts a more robust regulatory effect on the immune system by inducing Tregs and suppressing inflammatory cytokine production in Teffs in the intestines.

### 3.2. MPRO Treatment Increases the CD103^+^CD11b^+^ Regulatory Dendritic Cells in the Intestines

After confirming the immunomodulatory effects of MPRO administration, we delved into exploring the function of gut antigen-presenting cells in recognizing MPRO and eliciting anti-inflammatory responses. Given the pivotal role of dendritic cells (DCs) in shaping adaptive immune responses, we examined the intestinal DC populations in germ-free (GF) mice colonized with MPRO or its component strains. CD103-expressing DCs are crucial for the induction of regulatory T cells (Tregs) in the intestines, and these CD103^+^ DCs can be further categorized based on CD11b expression [27,28,29]. 

**Figure 2:**
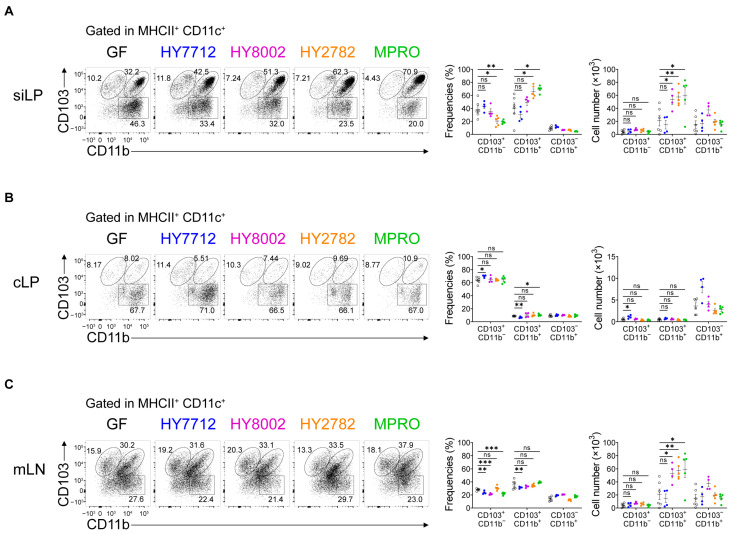
MPRO treatment increases regulatory CD103^+^CD11b^+^ dendritic cells in the intestines. GF C57BL/6 mice colonized with HY7712 (blue), HY8002 (magenta), HY2782 (orange), or MPRO (green) for 3 weeks were analyzed for alterations in the DC subtype population. GF mice were presented with black color open dots. Representative flow cytometry plots, frequencies, and absolute numbers of CD103^+^CD11b^−^, CD103^+^CD11b^+^, CD103^−^CD11b^+^ DCs within the MHCII^+^ CD11c^+^ cell population in the siLP (**A**), cLP (**B**), and mLN (**C**). All graphs show the mean ± SEM. * *p* < 0.05, ** *p* < 0.01, *** *p* < 0.001 (Two-tailed unpaired Student’s *t*-test). ns: not significant. Data were pooled from two independent experiments.

Intriguingly, within the small intestinal lamina propria (siLP), both the percentage and absolute cell number of CD103^+^CD11b^+^ DCs were significantly elevated following MPRO administration (Figure 2A). Although administration of HY2782 and MPRO, but not HY7712 and HY8002, decreased the frequencies of CD103^+^CD11b^−^ DCs in the siLP, the cellularity of these DCs was comparable to the germ-free (GF) group (Figure 2A). Additionally, within the colonic lamina propria (cLP), MPRO treatment did not show significant alterations in the frequency or number of CD103^+^CD11b^−^ DCs, while the proportion of CD103^+^CD11b^+^ DCs was slightly elevated with statistical significance (Figure 2B). We then analyzed the proportion of CD103^+^CD11b^+^ DCs in the mesenteric lymph nodes (mLN) following bacterial administration. Consistent with the small intestine, MPRO administration significantly increased the cellularity of CD103^+^CD11b^+^ DCs, but CD103^+^CD11b^−^ DCs were not altered (Figure 2C). Also, HY8002 and HY2782 administration could increase the absolute number of CD103^+^CD11b^+^ DC population in mLN (Figure 2C). We further examined the effect of individual bacteria and MPRO on macrophages in the gut. Since intestinal macrophages also play a role in shaping adaptive immunity in the intestine [30,31], we investigated the effect of bacterial administration on intestinal macrophages. Except for HY8002, none of the bacteria treatments significantly increased the frequencies and the absolute cell number of macrophages in both intestines and mesenteric lymph nodes (mLN) (Figure S2A,B). HY8002 treatment significantly elevated the cellularity of macrophages in the small intestine as well as mLN, although not affecting frequencies (Figure S2C). Collectively, MPRO is capable of increasing CD103^+^CD11b^+^ DCs within the small intestinal lamina propria (siLP) and mesenteric lymph nodes (mLN), rather than affecting macrophages, in exerting its systemic anti-inflammatory effects.

### 3.3. Administration of MPRO Confers Better Prophylactic Protection against Experimental Atopic Dermatitis Compared to Individual Bacterial Components

**Figure 3:**
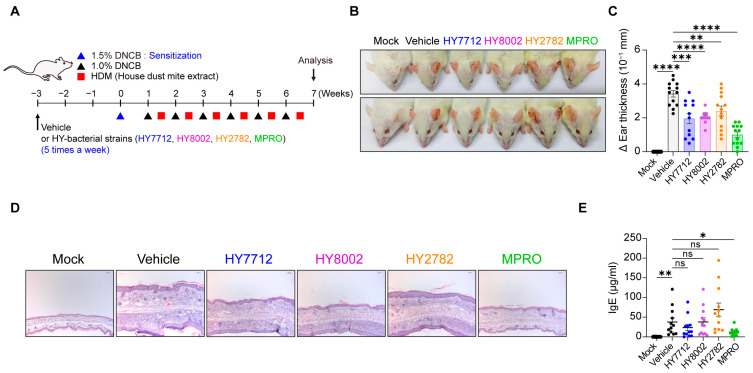
MPRO outperforms individual components in preventing atopic dermatitis. (**A**) Experimental scheme: BALB/c mice were pre-treated with vehicle, HY7712 (blue), HY8002 (magenta), HY2782 (orange), or MPRO (green) for three weeks. Mock group was presented with black color open dots and vehicle group was indicated with black closed dots. AD was induced by repeatedly painting DNCB and HDM on the ears for seven weeks alongside oral treatments. Inflammation in the ear (**B**), ear thickness (**C**), histological changes (H&E staining) (**D**), and serum IgE levels (**E**) were analyzed between the treatment groups. The graphs show the mean ± SEM. (**C**,**E**) * *p* < 0.05, ** *p* < 0.01, *** *p* < 0.001, **** *p* < 0.0001 (Two-tailed unpaired Student’s *t*-test). ns: not significant. Data pooled from two independent experiments.

To assess the efficiency of the consortium of strains compared to single bacterial strains in reducing inflammatory responses, we utilized two experimental disease models. First, we evaluated the immunoregulatory potential of the MPRO consortium and its individual component species in an experimental atopic dermatitis (AD) model. BALB/c mice were treated with MPRO, HY7712, HY8002, or HY2782 via oral gavage five times a week, starting three weeks before AD induction (Figure 3A). AD manifestations were monitored by measuring changes in ear thickness (Figure 3B,C), observing the structure of ear tissue (Figure 3D), and assessing immunoglobulin E (IgE) levels in the serum (Figure 3E). As expected, AD induction resulted in severe ear edema, significant increases in ear thickness, and the disruption of ear tissue structure (Figure 3B–D). Interestingly, all bacterial treatment groups improved AD manifestations, with MPRO demonstrating the most significant suppressive effects on ear tissue disruption and swelling (Figure 3B–D). Consistent with AD symptoms, serum IgE levels were elevated upon AD induction (Figure 3E). MPRO administration significantly suppressed serum IgE levels, while among the individual component strains, only HY7712 exhibited moderate, albeit nonsignificant, improvements (Figure 3E). In summary, the MPRO consortium displayed superior immunosuppressive potential under AD settings compared to its single component species.

### 3.4. MPRO Demonstrates a Heightened Regulatory Impact on the Immune System in Experimental AD

**Figure 4:**
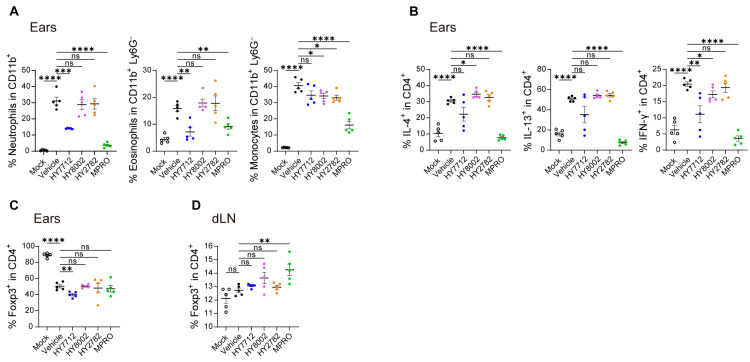
MPRO alleviates atopic dermatitis through anti-inflammatory immune regulation. Cells isolated from the ears or cervical draining lymph nodes of mice treated with vehicle, HY7712 (blue), HY8002 (magenta), HY2782 (orange), or MPRO (green) after 7 weeks of AD induction were analyzed. Mock group was presented with black color open dots and vehicle group was indicated with black closed dots. (**A**) Frequencies of neutrophil, eosinophil, or monocyte infiltration at inflamed ears of indicated mice. (**B**) Frequencies of IL-4^+^, IL-13^+^, or IFN-γ^+^ cells within the CD4^+^ T cell population. Frequencies of CD4^+^ Foxp3^+^ cells in the ears (**C**) and cervical draining lymph nodes (**D**) of indicated mice. All graphs show the mean ± SEM. * *p* < 0.05, ** *p* < 0.01, *** *p* < 0.001, **** *p* < 0.0001 (Two-tailed unpaired-Student’s *t*-test). ns: not significant. Data are representative of two to three independent experiments with similar results.

Under homeostatic conditions, MPRO demonstrates immunoregulatory effects by inducing Tregs and suppressing inflammatory cytokine expression in Teff cells (Figure 1). Next, we investigated whether MPRO could modulate the pathogenic immunological properties in the context of atopic dermatitis (AD). AD induction resulted in a significant increase in the frequencies of harmful inflammatory immune cells in the ear, comprising neutrophils, eosinophils, and monocytes (Figure 4A). Compared to individual bacterial components, MPRO substantially diminishes the infiltration of harmful inflammatory immune cells, such as neutrophils, eosinophils, and monocytes, in the ear tissues (Figure 4A).

HY7712 and MPRO administration were both capable of significantly reducing the neutrophil and eosinophil infiltration into the ear, with MPRO exhibiting the most pronounced effect (Figure 4A). MPRO treatment significantly reduced AD-associated monocyte populations in the ear, while HY8002 and HY2782 exhibited moderate improvements (Figure 4A and Figure S3A). Additionally, AD induction led to the elevated production of IL-4, IL-13, and IFN-γ cytokines by T cells in the ear (Figure 4B and Figure S3B). Individual component bacteria administration exhibited minimal differences, but MPRO treatment significantly suppressed all these T cell-derived inflammatory cytokines in the ear (Figure 4B and Figure S3B). We then examined the Treg population in the ear and draining lymph nodes (dLN). The frequency of Tregs in the ear of the MPRO group was comparable to that of the vehicle group (Figure 4C and Figure S3C). However, within the dLN of the ear, Tregs significantly increased with MPRO treatment (Figure 4D and Figure S3D). These findings suggest that MPRO alleviates AD symptoms by inducing Tregs and downregulating the pro-inflammatory response, as indicated by the reduced infiltration of inflammatory immune cells and suppressed production of inflammatory cytokines in the ear.

### 3.5. MPRO Alleviates Experimental Atopic Dermatitis by Reshaping the Microbiome Landscape 

**Figure 5:**
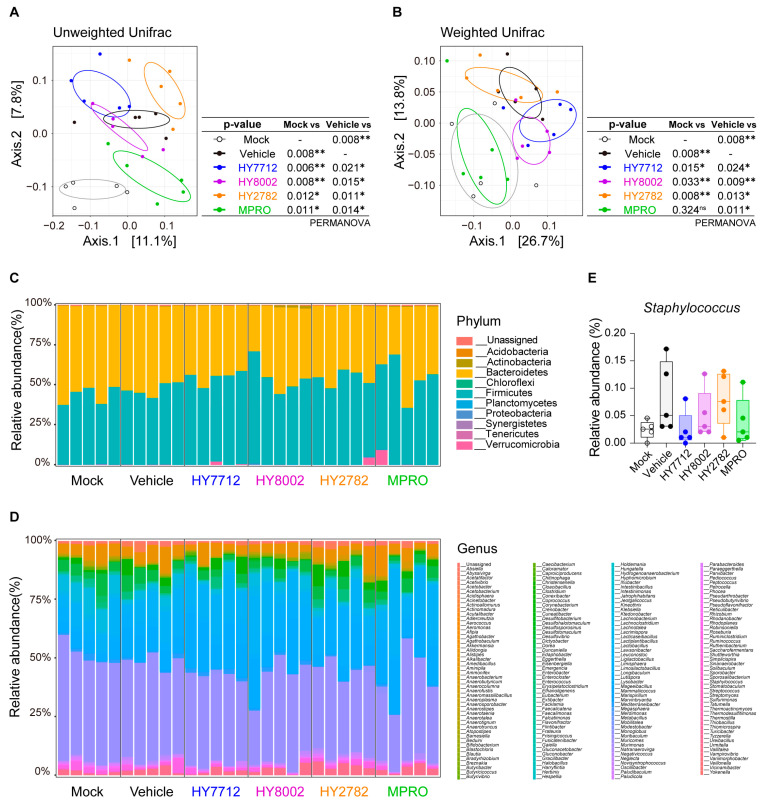
MPRO treatment modulates atopic dermatitis through microbiome modification. After administering vehicle, HY7712, HY8002, HY2782, or MPRO in AD−induced mice, stool samples were collected, and alterations in the microbiota were analyzed using Principal Coordinates Analysis of unweighted (**A**) and weighted (**B**) UniFrac distance based on 16s rRNA profiling of feces from indicated mice. * *p* < 0.05, ** *p* < 0.01 (PERMANOVA), ns: not significant. Relative abundance of indicated phylum-level (**C**) and genus-level (**D**) bacterial taxa in feces. (**E**) Relative abundance of *Staphylococcus*.

We delved into understanding how MPRO’s immunomodulatory effects differed from its individual bacterial components in the context of AD. Given the close association between microbial dysbiosis and AD, we conducted 16S rRNA sequencing analysis on fecal samples from AD mice treated with MPRO or its constituent bacteria. Principal Coordinate Analysis (PCoA) plots generated through beta-diversity analysis using UniFrac and weighted UniFrac distances revealed substantial alterations in the gut microbiome structure in AD-induced mice compared to their healthy counterparts (Figure 5A,B). While the administration of single bacterial strains in AD mice had minimal impact on the microbial composition, treatment with the MPRO consortium effectively reshaped the overall microbiome landscape, resembling that of a healthy microbiome (Figure 5A,B). This finding suggests that the MPRO consortium effectively mitigates AD-associated dysbiosis. 

A detailed analysis of the microbiota composition revealed significant alterations induced by AD, both at the phyla and genera levels. At the phylum level, there was an increasing trend in the relative abundance of *Firmicutes* and *Actinobacteria*, accompanied by a decreased tendency of *Bacteroidetes*, compared to AD mice without bacterial treatment (Figure 5C). On the genus level, MPRO administration led to elevated levels of *Bifidobacterium*, *Lismosilactobacillus*, and *Lactobacillus* (Figure 5D). Notably, previous studies have highlighted an elevated abundance of the *Staphylococcus* genus in AD patients, potentially contributing to AD pathophysiology [32,33,34]. In AD mice, *Staphylococcus* spp. were drastically elevated, with HY7712 and MPRO administration showing the ability to reduce their abundance (Figure 5E). Taken together, MPRO demonstrated its regulatory potential in AD by modifying the overall microbial landscape. Furthermore, the administration of MPRO’s individual component species only partially influenced the AD-associated microbiome, underscoring the synergistic potential of the MPRO consortium in shaping the microbial landscape toward that of the healthy control group.

### 3.6. MPRO Administration Ameliorates Experimental Inflammatory Colitis

**Figure 6:**
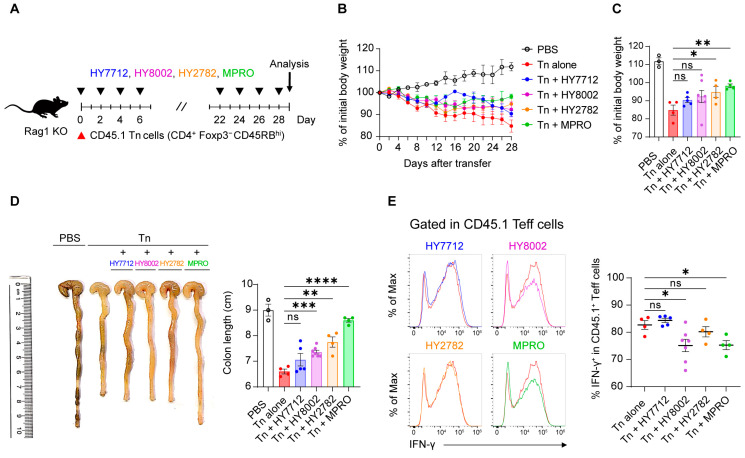
MPRO ameliorates the pathophysiology of experimental inflammatory colitis. (**A**) Experimental scheme: Naïve CD4^+^ T cells (CD45.1^+^CD4^+^Foxp3^−^CD45RB^hi^) were sorted and adoptively transferred into *Rag1* deficient mice. Naïve CD4^+^ T cells alone were transferred as controls (Tn alone group). Mice were treated with vehicle, HY7712 (blue), HY8002 (magenta), HY2782 (orange), or MPRO (green) every other day throughout the experiment. PBS group was presented with black open dots and Tn alone group was indicated with red dots. (**B**) Changes in body weight and (**C**) body weights at the end of the experiment (Day 28) were measured. (**D**) Representative image of colon and colon length. (**E**) Representative histograms and frequencies of IFN-γ producing colonic effector CD4^+^ T cells (CD45.1^+^CD4^+^Foxp3^−^). The graphs show the mean ± SEM. (**C**–**E**) * *p* < 0.05, ** *p* < 0.01, *** *p* < 0.001, **** *p* < 0.0001 (Two-tailed unpaired-Student’s *t*-test). ns: not significant. Data are representative of three independent experiments with similar results.

Following the demonstration of MPRO’s immunomodulatory capabilities in AD, we proceeded to evaluate its effectiveness in another inflammatory disorder model, colitis, which involves the inflammation of the colon. To induce experimental colitis, congenically labeled CD45.1^+^ naïve CD4 T cells were adoptively transferred into lymphopenic mice, specifically *Rag1*-deficient mice [35,36]. Subsequently, the mice received treatment with either MPRO or its individual component strains every other day throughout the experimental period (Figure 6A). Colitis induction resulted in body weight loss and a reduction in colon length (Figure 6B–D). While HY7712 and HY8002 showed only moderate to no protection from colitis, HY2782 and MPRO could prevent weight loss and colon shortening (Figure 6B–D). Measures of IFN-γ in donor cell-derived Teff cells from the colonic lamina propria (cLP) indicated that HY7712 and HY2782 showed no alterations, while only HY8002 and MPRO significantly reduced levels of this key inflammatory cytokine (Figure 6E). Collectively, the administration of MPRO’s individual strains demonstrated improvements in only specific aspects of colitogenic pathophysiology, but not in all three of our measures. Only MPRO conferred significant improvements in both body weights and colon lengths, along with a marked reduction in IFN-γ levels from Teff cells. Taken together, our data suggest that a consortium of bacteria is necessary for a synergistic immunoregulatory potential that can effectively alleviate the comprehensive pathophysiological manifestations of inflammatory colitis.

## 4. Discussion

Numerous studies have extensively explored the immunomodulatory properties of probiotic bacteria in health and disease [5,6,7,8,10,11,12]. However, most of the previous studies have focused on elucidating the immunomodulatory effects of individual bacterial species [7,37,38,39]. In contrast, fewer studies have examined the benefits of bacterial consortia [10,11,12,15]. Considerable advances in understanding the gut–immune axis during the recent few decades have brought immunoregulatory bacteria to the spotlight, with the intent of using them in the development of next generation therapeutics against diverse inflammatory diseases or autoimmunity. However, unmet questions persist regarding the most effective methodology for developing microbiome-based therapeutics, specifically whether single strains or bacterial consortia are superior against inflammatory disorders. 

In our study, we comprehensively compared the immunomodulatory potentials of a bacterial consortium and its individual single-strain components, both in healthy and diseased conditions, evaluating their impacts on the gut microbial landscape. Our results unequivocally demonstrate the superior efficacy of bacterial consortia against inflammatory disorders compared to single-strain bacteria. This enhanced efficacy arises from multiple mechanisms, including reshaping the microbiome landscape and inducing anti-inflammatory responses mediated by regulatory dendritic cells (DCs) and Treg cells.

In our previous studies, we specifically studied the MPRO consortium and its individual component strains in different contexts without comparison between the single stain and MPRO consortium. For instance, HY2782 and HY8002 have shown efficacy in mitigating airway hyperresponsiveness and pulmonary inflammation induced by chronic exposure to fine particulate matter (PM_2.5_) through the intranasal route [40]. In addition, our study is one of the few to provide clinical relevance for building a consortium of multiple bacterial species for effective therapeutics. We previously demonstrated the clinical efficacy of MPRO. The administration of MPRO has benefited colon cancer patients recovering from anterior resection surgery, restraining postoperative bowel dysfunction and reducing inflammatory signatures [41]. More specifically, compared to the placebo cohort, the administration of MPRO to patients relatively restrained the exacerbation of postoperative bowel dysfunction, which was evaluated by measurements of anterior resection syndrome (ARS) and the capability for flatus control [41]. In addition, MPRO supplementation alleviated inflammatory signatures after the surgery and moderately, although not statistically significantly, decreased the relative abundance of several deleterious bacterial species associated with colon cancer [41]. In line with previous findings, including clinical applications, our study demonstrates that MPRO, compared to its individual component strains, significantly alleviates clinical symptoms associated with various inflammatory disorders. These anti-inflammatory effects of MPRO depend on two distinct mechanisms, one involving the modulation of the microbiota composition and the second shaping the immune system. Furthermore, our data demonstrate that using single strains of bacteria not only has relatively low efficacy against inflammatory diseases but also an insufficient capability to modulate the microbiota landscape compared to their consortium. For instance, in the AD settings, while HY7712 could reduce neutrophil and eosinophil infiltration into the ear and improve AD outcomes, it could not protect against experimental colitis. Meanwhile, HY8002 could reduce IFN-γ production and protect from colon shortening during experimental colitis; still, it could not prevent body weight loss in the experimental colitis model and confer partial protection from AD. Likewise, HY2782 showed effective IL-10^+^ and RORγt^+^ Helios^−^ Treg induction in the siLP and cLP during healthy conditions but failed to protect mice against AD and colitis effectively. The mixed effectiveness of each strain during different conditions and the combined efficacy of the MPRO consortium indicate a synergistic output suitable for a wider range of inflammatory disorders.

When administered to GF mice, MPRO exerted regulatory effects on the intestinal immune system. Under healthy conditions, MPRO enhanced bacteria-induced Treg cells and IL-10 production in the intestines while repressing effector T cells and inflammatory cytokines. Furthermore, under atopic dermatitis conditions, MPRO significantly restrained detrimental inflammatory immune responses, including reduced ear swelling, suppressed immune cell infiltration, and decreased serum immunoglobulin E levels. These effects are likely attributed to the upregulation of intestinal regulatory CD103^+^CD11b^+^ dendritic cells and alterations in the microbiome communities. Intestinal CD103^+^CD11b^+^ dendritic cells serve as pivotal regulators of immune responses by fostering the generation of intestinal Treg cells, both in states of health and during disease [42]. These dendritic cells, present in the intestinal lamina propria, adeptly capture luminal antigens and subsequently migrate to the mesenteric lymph nodes, where they actively promote the differentiation of Treg cells [7,43].

However, the precise molecular mechanisms behind MPRO’s immunoregulatory properties, particularly through dendritic cells, remain unclear. Investigating these mechanisms in-depth could enhance the efficient utilization of MPRO for additional clinical therapeutics. Further studies exploring the molecular basis of the synergistic effects of bacterial consortia are essential to develop strategies for selecting specific strains for inclusion. Our study’s limitation lies in the composition of MPRO, which includes only three bacterial species. Given the multitude of bacteria with reported immunomodulatory functions and the ability to alter the microbial landscape, future research involving larger, more diverse consortia is necessary to develop effective microbiome-based therapeutics against various inflammatory and allergic disorders.

## 5. Conclusions

In conclusion, our study underscores the significant immunomodulatory potential of MPRO, a consortium of bacteria, in mitigating inflammatory disorders such as atopic dermatitis (AD) and colitis. By modulating immune cell populations, inducing the regulatory T cells, suppressing inflammation, and reshaping of the gut microbiome, MPRO exhibited superior efficacy compared to its individual component strains. The findings highlight the importance of a consortium approach in achieving synergistic effects against inflammatory conditions, indicating MPRO as a promising candidate for future therapeutic interventions in such disorders.

## Data Availability

The 16S rRNA sequencing data were deposited in the National Center for Biotechnology Information (NCBI)’s Sequence Read Archive (SRA) under BioProject accession PRJNA1056948.

## Supplementary Materials

The following supporting information can be downloaded at: https://www.mdpi.com/article/10.3390/nu16060790/s1, Figure S1: Effect of bacteria treatment on Treg cells in the mLN; Figure S2: Effect of bacteria treatment on CX3CR1^+^ macrophages; Figure S3: MPRO treatment ameliorates experimental AD by suppressing inflammatory immune responses; Table S1: List of antibodies used in flow cytometry.
