# Supplementary material for: Probiotic Consortium Confers Synergistic Anti-Inflammatory Effects in Inflammatory Disorders
Source: Nutrients. 2024 Mar 11;16(6):790. doi: 10.3390/nu16060790 (PMC10975258; doi:10.3390/nu16060790)
Supplement: Supplementary file 1 [file nutrients-16-00790-s001.zip › Supplementary_Nutrients.pdf]

Supplementary Material

Probiotic Consortium Confers Synergistic Anti-inflammatory Effects in Inflammatory Disorders

Changhon Lee [1,2,+], Seung Won Kim [1,+], Ravi Verma [3,4], Jaegyun Noh [1], John Chulhoon Park [1], Sunhee Park [3,5], Haena Lee [1], Hye Eun Park [1], Chan Johng Kim [1,6], Seohyun Byun [1,7], Haeun Ko [1], Seungyeon Choi [3], Inhae Kim [3], Soomin Jeon [8], Junglyoul Lee [8,*] and Sin-Hyeog Im [1,3,9,*]

[1] Department of Life Sciences, POSTECH Biotech Center, Pohang University of Science and Technology (POSTECH), Pohang, 37673, Republic of Korea
[2] Current address: Department of Immunology, Blavatnik Institute, Harvard Medical School, Boston, MA, 02115, USA
[3] ImmmunoBiome Inc, 77 Cheongam-Ro, Nam-Gu, Pohang, 37673, Republic of Korea
[4] Current address: School of Biotechnology, Jawaharlal Nehru University, New Delhi 110067, India
[5] Current address: Natural Product Informatics Research Center, Korea Institute of Science and Technology (KIST), Gangneung, 25451, Republic of Korea
[6] Current address: Department of Biochemistry and Institute for Protein Design, University of Washington, Seattle, WA, 98195, USA
[7] Current address: Celltrion, Inc, 22 Academy-ro 51beon-gil, Yeonsu-gu, Incheon, 22014, Republic of Korea
[8] hy Co. Ltd, 22 Giheungdanji-Ro 24 beon-gil, Giheung-Gu, Yongin, 17086, Republic of Korea
[9] Institute for Convergence Research and Education, Yonsei University, Seoul, 03722, Republic of Korea
+ Equal contributors
* Correspondence: junyeop0315@naver.com (J.L.); iimsh@postech.ac.kr (S.H.I)

Supplementary Figures

Figure S1

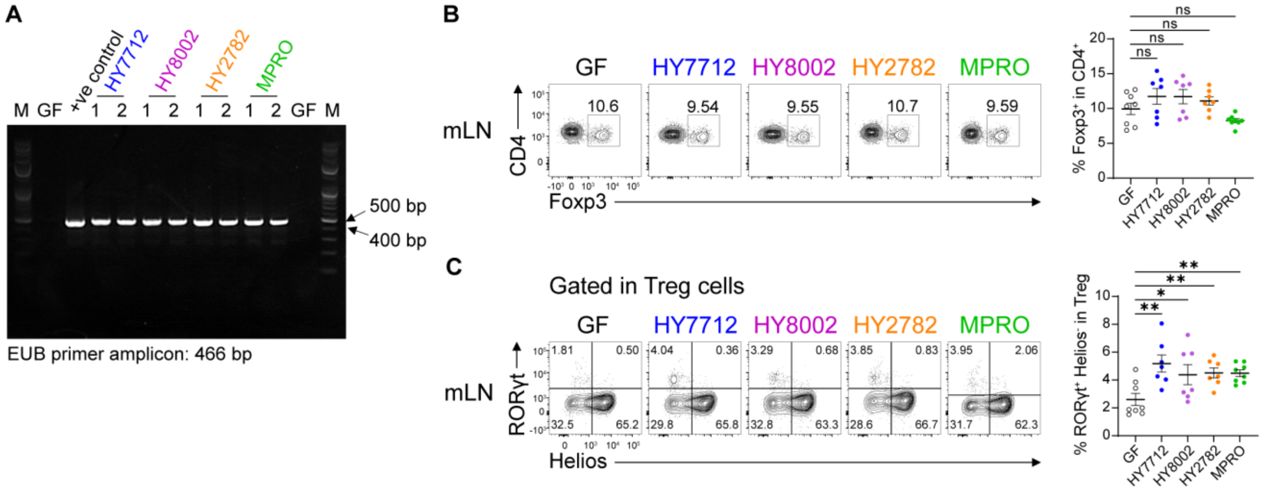

Figure S1. Effect of bacteria treatment on Treg cells in the mLN. (**A**) PCR analysis was conducted to confirm the colonization of germ-free (GF) mice after treatment with indicated bacterial strains. DNAs isolated from GF mice colonized with respective bacteria were amplified using EUB universal primers and loaded onto an agarose gel with a marker for analysis. (**B**) Representative flow cytometry plots and frequencies of Foxp3⁺ cells within the CD4⁺ T cell population in the lamina propria of mesenteric lymph node (mLN) of the indicated mice. (**C**) Representative flow cytometry plots and frequencies of RORγt⁺Helios⁻ cells within the Treg cell population in the mLN of the indicated mice. The graphs show the mean ± SEM. (**B**, **C**) $*p < 0.05$, $**p < 0.01$ (Student's t test). ns: not significant. Data pooled from two to three independent experiments.

Figure S2

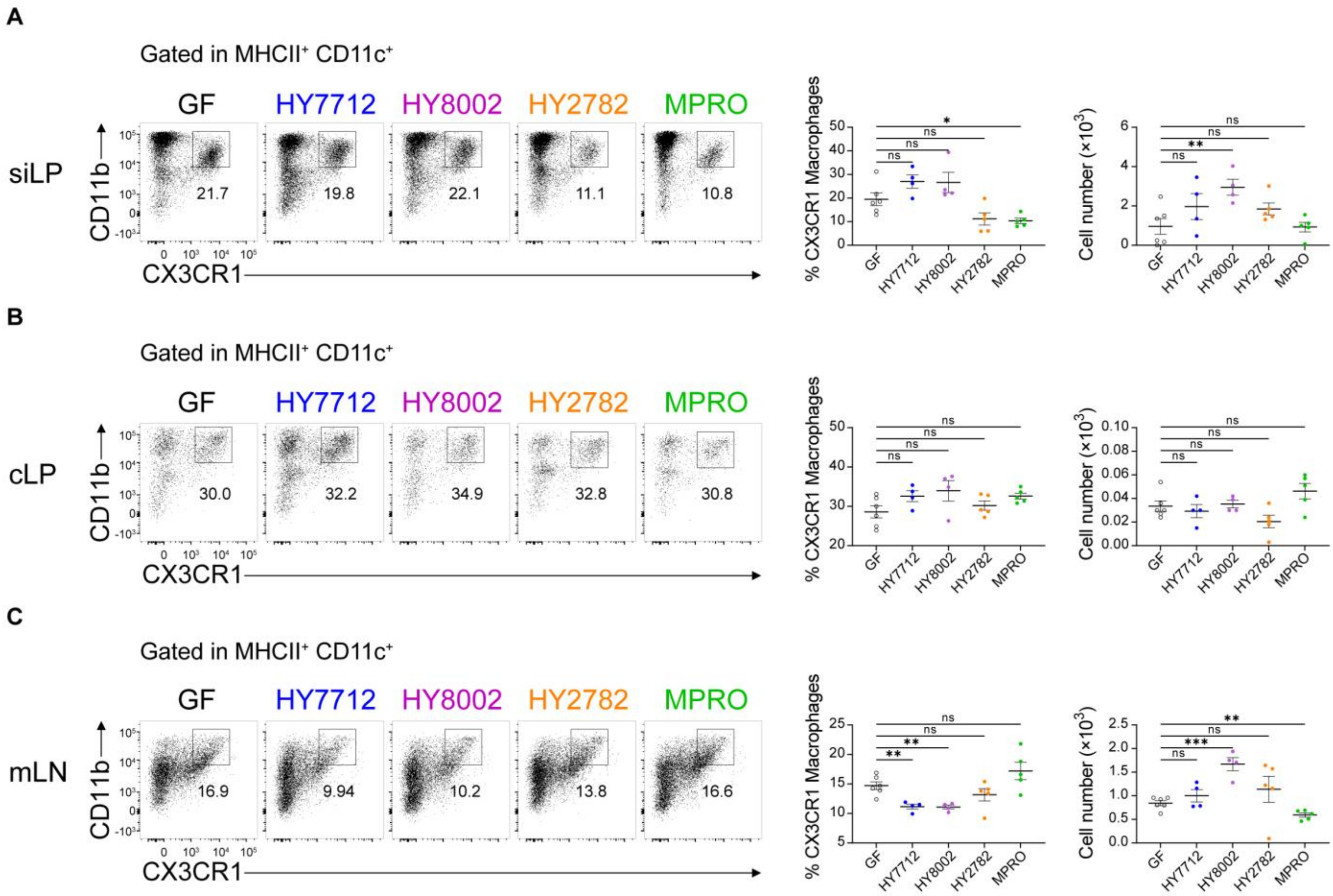

Figure S2. Effect of bacteria treatment on CX3CR1⁺ macrophages. GF C57BL/6 mice were colonized with HY7712, HY8002, HY2782, or MPRO for 3 weeks, and CX3CR1⁺ macrophages within the MHCII⁺ CD11c⁺ cell population were analyzed by flow cytometry in the siLP (**A**), cLP (**B**) and mLN (**C**). All graphs show the mean ± SEM. *$p < 0.05$, **$p < 0.01$, ***$p < 0.001$ (Student's t test). ns: not significant. Data pooled from two independent experiments.

Figure S3

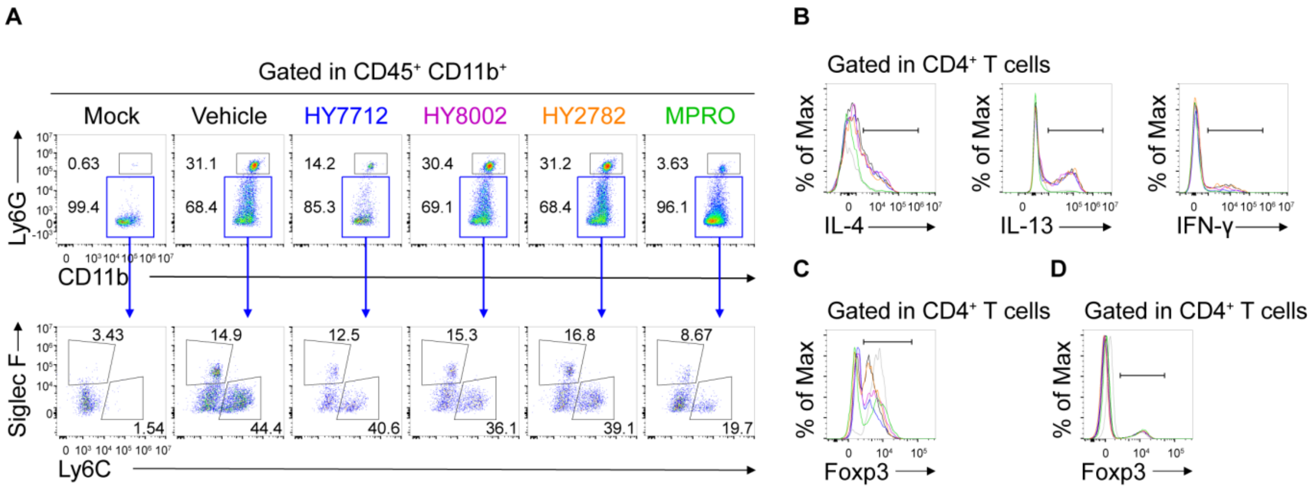

Figure S3. MPRO treatment ameliorates experimental AD by suppressing inflammatory immune responses. Cells were isolated from mice's ears or cervical draining lymph nodes treated with vehicle, HY7712, HY8002, HY2782, or MPRO after 7 weeks of AD induction. (**A**) Representative flow cytometry plots of neutrophil, eosinophil, or monocyte infiltration at inflamed ears of indicated mice. (**B**) Representative flow cytometry plots of IL-4⁺, IL-13⁺, or IFN-γ⁺ cells within the CD4⁺ T cell population in the ears of indicated mice. Representative histograms of Foxp3⁺ cells within the CD4⁺ T cell population in the ears (**C**) and cervical draining lymph nodes (**D**) of indicated mice.

Supplementary Tables

Supplementary Table S1. List of antibodies used in flow cytometry

Target	Conjugate	Source	#Catalog	Clone
CD45	BV605	Biolegend	103140	30-F11
CD45.1	APC	Biolegend	110714	A20
TCRβ	A488	Biolegend	109215	H57-597
TCRβ	APC	Biolegend	109212	H57-597
MHCII	APC/Cy7	Biolegend	107628	M5/114.15.2
CD11b	PE/Cy7	Biolegend	101216	M1/70
CD11b	PerCP/Cy5.5	TONBO	65-0112-U100	M1/70
B220	FITC	TONBO	35-0452-U100	RA3-6B2
CD4	BUV395	BD bioscience	563790	GK1.5
CD4	BV605	Biolegend	100548	RM4-5
CD8a	APC	TONBO	20-0081-U100	53-6.7
CD8a	PerCP/Cy5.5	TONBO	65-0081-U100	53-6.7
CD8a	APC/Cy7	TONBO	25-0081-U100	53-6.7
CD8a	BUV395	BD bioscience	563786	53-6.7
CD11c	FITC	TONBO	35-0114-U100	N418
Ly6C	BV510	Biolegend	128033	HK1.4
Ly6G	APC	Biolegend	127613	1A8
Siglec F	PE	BD Bioscience	552126	E50-2440
CD103	BV421	Biolegend	121422	2E7
CX3CR1	PE	Biolegend	149006	SA011F11
CD317	APC	Biolegend	127016	927
Nrp1	PerCP/Cy5.5	Biolegend	145208	3E12
CTLA-4	BV421	Biolegend	106312	UC10-4B9
CD45RB	PE	BD Bioscience	553101	16A
Foxp3	PE/Cy7	Invitrogen	25-5773-82	FJK-16s
Foxp3	FITC	Invitrogen	11-5773-82	FJK-16s
T-bet	PE/Cy7	Invitrogen	25-5825-82	eBio4B10 (4B10)
GATA-3	BV421	Biolegend	653814	16E10A23
RORγt	PE	Invitrogen	12-6981-82	B2D
Helios	A488	Biolegend	137223	22F6
IFN-γ	PerCP/Cy5.5	Biolegend	505822	XMG1.2
IFN-γ	PE	Invitrogen	12-7311-82	XMG1.2
IFN-γ	APC	Invitrogen	17-7311-82	XMG1.2
IL-10	BV421	Biolegend	505022	JES5-16E3
IL-13	PerCP/eF710	Invitrogen	46-7133-82	eBio13A

Continued in next page.

Supplementary Table S1. List of antibodies used in flow cytometry

Target	Conjugate	Source	#Catalog	Clone
IL-17A	APC	Biolegend	506916	TC11-18H10.1
IL-17A	PE/Cy7	Biolegend	506922	TC11-18H10.1
IL-17A	PE	Biolegend	506904	TC11-18H10.1
IL-4	PE	Invitrogen	12-7041-81	11B11
IL-4	PE/Cy7	Invitrogen	25-7041-82	11B11
FVD	eF506	Invitrogen	65-0866-18	
FVD	eF780	Invitrogen	65-0865-18	
